# Sleep attitudes as a predictor of sleep outcomes: a secondary data analysis

**DOI:** 10.1080/21642850.2020.1852939

**Published:** 2020-12-08

**Authors:** Aria R. Ruggiero, Hannah D. Peach, Philip Zendels, Jane F. Gaultney

**Affiliations:** Department of Psychological Sciences, University of North Carolina at Charlotte, Charlotte, NC, USA

**Keywords:** Sleep attitudes, health disparities, sleep health, sleep hygiene, moderated mediation

## Abstract

**Objectives:**

Attitudes have been widely studied as predictors of a number of social and health behaviors. However, attitudes predicting sleep outcomes have only recently been examined, despite sleep being conceptualized as an important health behavior. Prior research has demonstrated that attitudes toward sleep are associated with sleep hygiene, sleep duration and quality (Peach & Gaultney, 2017; Peach, Gaultney, Ruggiero, 2018). Sleep attitudes interact with varying demographic identities, such as age, gender, race, and perceived socioeconomic status (SES) (Ruggiero, Peach, & Gaultney, 2019). The present study hypothesized that (1) sleep attitudes would be indirectly associated with sleep outcomes (duration and quality) via sleep hygiene, and, (2) this indirect effect would be modified by specific demographic variables (age, gender, race, and perceived SES; moderated mediation).

**Method:**

One hundred and seventy-two adults from the United States completed an anonymous survey on sleep characteristics and health.

**Results:**

Results confirmed the first hypothesis, indicating that sleep attitudes were significantly and indirectly associated with both sleep duration and sleep quality via sleep hygiene. Additionally, gender and SES further modified these significant indirect effects, meaning hypothesis two was partially supported.

**Conclusions:**

Results are discussed in terms of their implications for the importance and variability of sleep attitudes, and future research directions are considered.

## Demographic disparities in health

There are many facets of health (e.g. diet, exercise, sleep) that are predictive of an individual’s overall quality of health. However, differences in health often exist as a function of demographic characteristics, such as age, gender, race, and socioeconomic status (SES), which highlights how aspects of health, including attitudes, practices, and outcomes, are shaped by many other variables. For instance, older age and identifying as a woman have been associated with more positive help-seeking attitudes, which is related to utilization of health care services, including seeking mental health services (Mackenzie, Gekoski, & Knox, 2006). These gender differences in health attitudes and utilization have in part been attributed to a masculinity ideology, which may deter seeking care when needed (Wade, 2008). Moreover, the constraints that affect everyday choices and opportunities to make health a priority may vary for men and women (Rieker & Bird, 2005).

Additionally, general racial/ethnic disparities in healthcare exist in quality of care, morbidity and mortality. Identifying as a racial minority has been linked to earlier onset of illness and more severe illnesses in comparison to Whites (e.g. Williams, Mohammed, Leavell, & Collins, 2010). Moreover, white individuals have demonstrated more favorable attitudes than racial minorities toward new medical drugs, devices, and procedures (Groeneveld, Sonnad, Lee, Asch, & Shea, 2006). Prior research on racial disparities in health and health care suggest that minority individuals tend to have poorer overall health outcomes, fewer protective health behaviors, and worse access to quality health care, compared to White individuals (Dubay & Lebrun, 2012; Smedley, Stith, & Nelson, 2003). However, it has been argued that racial differences in health and health care may be due more to socioeconomic and social factors than to race, per se.

Health disparities based on socioeconomic status have also been documented in the literature. People with lower income and lower levels of education have worse health behaviors (e.g. tobacco use, physical inactivity, poor nutrition), health outcomes (e.g. cardiovascular health, overall mortality), and health care (e.g. access to and quality of health care), compared to people with higher income and higher education (Pampel, Krueger, & Denney, 2010). Additionally, lower SES has been associated with less health consciousness (i.e. thinking about things to do to keep healthy), stronger beliefs in the influence of chance on health, less thinking about the future, and lower life expectancies. Moreover, these attitudinal factors were in turn associated with unhealthy behavioral choices, independent of age, sex, and self rated health (Wardle & Steptoe, 2003).

However, less attention overall has been paid to disparities based on SES (Dubay & Lebrun, 2012). This is problematic considering that evidence suggests SES is perhaps a stronger determinant of health-related outcomes than race, with several studies demonstrating that the effect of race/ethnicity on health outcomes diminishes significantly or altogether disappears when SES is controlled (Williams, 1996). Therefore, while there is a significant body of research highlighting racial disparities in health, it is just as important to focus on socioeconomic disparities, as well, and to control for the relative contribution of each when examining potential health outcomes.

## Sleep and demographic differences

Sleep outcomes, like general health outcomes, also vary based on demographic characteristics, such as age, gender, race, and socioeconomic status, although mixed findings make it difficult to draw conclusions. For instance, some studies demonstrated worse sleep latency and a decrease in REM sleep with increasing age (Ohayon, Carskadon, Guilleinault, & Vitiello, 2004), while other studies found an increase in sleep quality and duration in older adults (Grandner et al., 2012). Similarly, some research reported that women tended to have better sleep quality (Krishnan & Collop, 2006), while in other studies women reported poorer sleep quality (Reyner & Horne, 1995). Research on disparities in sleep based on race/ethnicity and SES has been somewhat more consistent. Racial minorities, such as African Americans, have longer sleep latency, report poorer sleep quality, and obtain less deep sleep than white individuals (Durrence & Lichstein, 2006; Petrov & Lichstein, 2016). Moreover, low SES has been linked with higher rates of sleep disturbances, including difficulty falling or staying asleep, and shorter sleep duration (Grandner et al., 2013). Despite these findings, the degree to which race and SES uniquely contribute to facets of sleep remains uncertain (Mezick et al., 2008), and this is important to tease apart considering that race/ethnicity tends to highly converge with SES (Jackson & Williams, 2006).

## Sleep attitudes

Attitudes are viewed as modifiable predictors of a number of social and health behaviors, such as sun-protective behavior (Prentice-Dunn, McMath, & Cramer, 2009), and smoking cessation (Rise, Kovac, Kraft, & Moan, 2008). Thus, attitudes are one component that can shape health behaviors and outcomes. Less is known about the role of attitudes towards sleep on sleep hygiene and sleep outcomes. Some prior research has defined dysfunctional attitudes towards sleep as ‘psychological conceptu­alization of insomnia,’ or ‘faulty beliefs and attitudes about sleep’ (Morin, Vallières, & Ivers, 2007, p. 1547) as measured via the Dysfunctional Beliefs and Attitudes about Sleep Scale (DBAS-16; Morin et al., 2007). The DBAS has been shown to predict sleep and sleep hygiene in young adults (Yang, Chou, & Hsiao, 2011) and college students (Woodley & Smith, 2006). Yet, these definitions are limited, as sleep is both a health behavior and a biological state of consciousness that is necessary for survival, therefore a more comprehensive conceptualization is necessary for defining sleep attitudes. Thus, based on the work of Eagly and Chaiken (2007), the present study defined sleep attitudes as the propensity to evaluate sleep with some degree of favor or disfavor that is formed, informed, and expressed by cognitive, affective, and behavioral processes (Peach & Gaultney, 2017).

## The present study

Previous work has found a link between sleep attitudes and sleep outcomes (duration and quality) (Ruggiero, Peach, & Gaultney, 2019) and importantly, has shown that this relationship also functions indirectly via sleep hygiene (Peach, Gaultney, & Ruggiero, 2018; Ruggiero et al., 2019), with more favorable sleep attitudes relating to better sleep hygiene, which, in turn, predicted better sleep outcomes. Moreover, the association between sleep attitudes and sleep outcomes varied as a function of age, gender, race, and SES, with older adults, women, and white participants reporting more favorable sleep attitudes, while race made little difference in sleep attitudes among those with lower SES, but was more pronounced among those with higher SES (Ruggiero et al., 2019). Furthermore, female minority participants with less positive sleep attitudes reported the least amount of sleep on weekends, and high SES males with less favorable sleep attitudes reported more weekend sleep. Women with high SES and less favorable sleep attitudes received the least amount of sleep on weekends, indicating that having a higher SES may not always be beneficial for positive health behaviors like sleep, particularly for women (Ruggiero et al., 2019).

Therefore, the present study built on existing findings among college students that sleep attitudes predicted sleep hygiene and sleep outcomes (i.e. duration and quality; Peach et al., 2018) and aimed to extend the external validity of these findings among a broader sample of adults by using secondary data collected from an adult sample within the United States as compared to only sampling college students. This serves two goals: it allows for replication of earlier findings and extends the generalizability of those earlier findings by Peach et al. (2018) by exploring whether sleep attitudes predict sleep outcomes indirectly via sleep hygiene in an older, more diverse sample of adults rather than college students. Hypothesis one predicted a significant indirect effect between sleep attitudes and sleep outcomes via sleep hygiene in a more general adult sample. Earlier work also reported demographic differences in sleep attitudes (Ruggiero et al., 2019). The present study expanded consideration of demographic moderators by analyzing whether the indirect effect was modified by demographic characteristics. Therefore, the second goal was to test a moderated mediation model (Hayes, 2018).

## Method

The present study is a secondary data analysis of Ruggiero et al. (2019). Please refer to the original paper for details on the study’s sample and methodology. Table 1 contains demographic and descriptive data for the present study’s sample.

**Table 1.** Bivariate Correlations Between Predictors and Outcome Variables.

	%(N)	M(SD)	1	2	3	4	5	6	7
1. Age		33.31(9.86)	–						
2. Gender^a^ (% female)	41(70)		.23**	–					
3. Race^b^ (self-identified minority)	32(54)		.19*	−.02	–				
4. SES		4.76(1.76)	.07	−.12	.07	–			
5. Sleep Attitudes		5.14(.80)	.24**	.21**	.18*	.02	–		
6. Sleep Hygiene^1^		78.21(24.41)	−.10	−.07	−.22**	−.04	−.58**	–	
7. Sleep Duration^2^		7.35(1.47)	−.07	−.11	.14	.11	.18*	−.20**	–
8. Sleep Quality^3^		5.55(3.78)	−.01	.11	−.16*	−.05	−.35**	.60**	−.49**

Note: ^1^Higher scores indicate *poorer* sleep hygiene; ^2^Higher scores indicate *longer* sleep duration; ^3^Higher scores indicate *poorer* sleep quality. A score of 5 or greater for sleep quality indicates a ‘poor’ sleeper.

*p < .05; **p < .01. ^a^Males = 0, Females = 1; ^b^Self-identified racial/ethnic minority = 0, Self-identified white = 1. Correlations between dichotomous (race, gender) and continuous variables are point-biserial.

### Materials

Participants reported demographic variables including age, gender, race, perceived SES, use of medication that could affect sleep, and history of sleep disorder diagnosis. Sleep attitudes, sleep hygiene, and self-reported sleep duration and quality were also measured using psychometrically sound scales. For a full description of these scales, please refer to the original paper, Ruggiero et al., 2019, that this secondary data analysis refers to.

### Procedure

Participants completed an online questionnaire via the website Qualtrics (www.qualtrics.com), which asked participants questions regarding demographic information, perceived stress, exercise, eating habits, sleep attitudes, sleep hygiene, and sleep outcomes. Participants were presented with a screen with a consent statement, acknowledging that they were at least 21 years of age and living in the United States, and they indicated their consent by agreeing to these terms and continuing on to the survey questions. The institutional review board of the university at which this study was conducted approved the present study.

### Plan of analysis

Data on race were collected via an open-ended prompt and were manually dummy coded and dichotomized by two researchers to ensure accuracy and inter-rater reliability. Gender and race were dummy coded, (0 = males, racial minority), while age and SES were left continuous. One participant who identified as neither male nor female was excluded from the sample due to the small number, leaving 172 participants in the analysis.

After examining initial descriptive statistics and correlations using SPSS 25.0 (IBM Corporation, Armonk NY, USA), the test of the first hypothesis used a path model to examine sleep attitudes as the predictor, sleep hygiene as the indirect pathway, and sleep duration and sleep quality (separate analyses, each controlling for the other outcome in order to consider unique contributions of each aspect of sleep) as outcome variables (Hypothesis 1).

**Figure 1.**
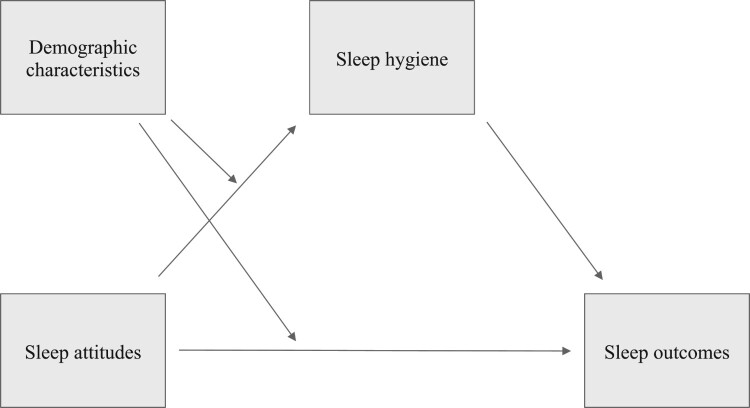
Proposed moderated mediation model of the indirect effect of sleep attitudes on sleep outcomes via sleep hygiene, with demographic variables moderating the direct effect of sleep attitudes on sleep hygiene and the indirect effect of sleep attitudes on sleep outcomes (Hayes, 2018, Model 8).

Following this, we used conditional process modeling to test whether the direct or indirect effect of sleep attitude was moderated by demographic variables, using the PROCESS macro (Hypothesis 2, Moderated Mediation model 8; Hayes, 2018). Specifically, we examined whether direct or indirect effects were moderated by gender, race, age, or SES (four analyses, each with a different moderator, for each of two sleep outcomes). See Figure 1 for a representation of the proposed model. Each model used the remaining sleep outcome and moderators as covariates, and 10,000 bootstrap samples confirmed confidence intervals. All results are reported using the bootstrapped 95% confidence interval from the index of moderated mediation, which was developed by Hayes (2015) to provide the most direct test for evidence of moderated mediation.

## Results

Correlations between predictor and outcome variables were in the expected directions (see Table 1). Tables 2 and 3 include direct, indirect, and moderated mediation coefficients for each analysis. Sleep duration and quality results are reported separately for each moderator.

**Table 2.** Test of Moderated Mediation Effect for Sleep Duration.

Moderator variable	Conditional indirect effects	*Effect*	SE	BootLLCI	BootULCI
Age					
	25 years (−1 SD)	−.15	.07	−.31	−.02
	31 years (Median)	−.13	.06	−.27	−.02
	42 (+1 SD)	−.10	.05	−.22	−.01
	Age Index	.01	.01	−.01	.01
Gender					
	Males	−.15	.08	−.31	−.01
	Females	−.08	.05	−.19	.01
	Gender Index	.07	.06	−.01	.20
Race					
	Racial/ethnic minorities	−.14	.07	−.29	−.03
	Whites	−.14	.07	−.29	−.03
	Race Index	.01	.05	−.10	.09
SES					
	Lower SES (−1 SD)	−.10	.05	−.21	−.01
	Median SES	−.13	.06	−.27	−.02
	Higher SES (+1 SD)	−.18	.08	−.35	−.02
	SES Index	−.02*	.01	−.05	−.001

Note: *N* = 172. *p < .05. *Effect* = unstandardized regression coefficient; Standard errors and bootstrapped confidence interval limits (BootLLCI, BootULCI) have been biased-corrected with a 10,000-resample bootstrap method (see Hayes, 2018).

**Table 3.** Test of Moderated Mediation Effect for Sleep Quality.

Moderator variable	Conditional indirect effects	*Effect*	SE	BootLLCI	BootULCI
Age					
	25 years (−1 SD)	−1.36	.28	−1.95	−.86
	31 years (Median)	−1.24	.23	−1.71	−.83
	42 (+1 SD)	−1.02	.26	−1.56	−.53
	Age Index	.02	.02	−.02	.06
Gender					
	Males	−1.49	.26	−2.02	−1.01
	Females	−.78	.26	−1.32	−.27
	Gender Index	.71*	.31	.14	1.36
Race					
	Racial/ethnic minorities	−1.31	.30	−1.91	−.71
	Whites	−1.19	.26	−1.74	−.73
	Race Index	.11	.34	−.59	.75
SES					
	Lower SES (−1 SD)	−.93	.26	−1.47	−.46
	Median SES	−1.26	.21	−1.71	−.87
	Higher SES (+1 SD)	−1.59	.27	−2.11	−1.06
	SES Index	−.17*	.08	−.31	−.01

Note: *N* = 172. *p < .05. *Effect* = unstandardized regression coefficient; Standard errors and bootstrapped confidence interval limits (BootLLCI, BootULCI) have been biased-corrected with a 10,000-resample bootstrap method (see Hayes, 2018).

**Figure 2.**
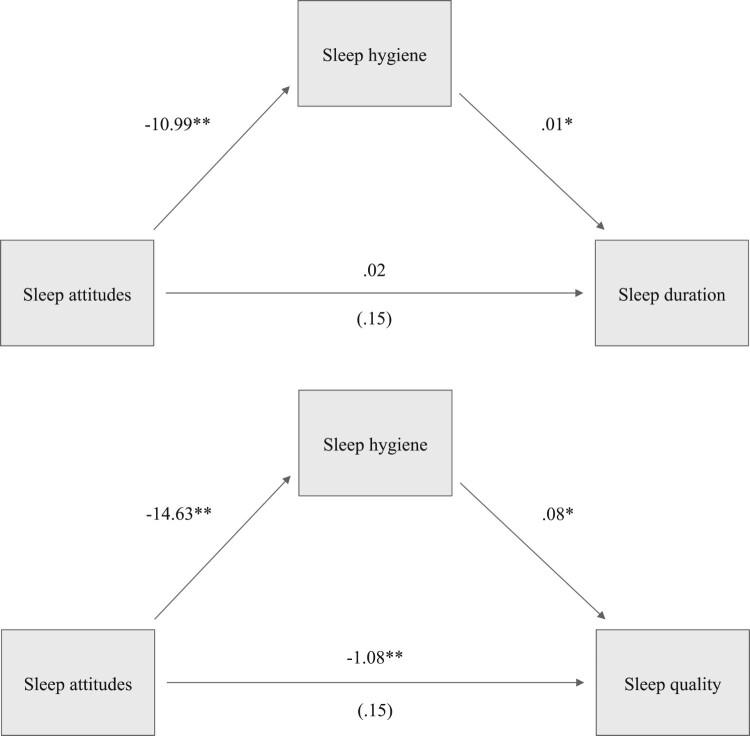
N = 172, *p < .05, **p < .01. Path models highlighting the relationship between sleep attitudes and sleep outcomes (duration and quality) via sleep hygiene. Coefficients for the c and c’ paths represent the total effect and direct effect of IV on DV, respectively. The direct effect is provided in parentheses.

Hypothesis one proposed an indirect relationship between sleep attitudes and sleep outcomes via sleep hygiene practices. Results demonstrated that this was indeed true for both sleep duration (effect = −.13, 95% CI [−.29, −.02]) and sleep quality (effect = −1.24, 95% CI [−1.71, −.85]) (see Figure 2). Thus, previously reported associations found among college students were replicated among an older, more diverse sample of adults.

Next, we hypothesized that the indirect effect of sleep attitudes via sleep hygiene would be contingent on varying levels of the moderator variable (i.e. age, gender, race, and SES), producing a moderated mediation effect. Thus, there would be a conditional effect of the moderator on both the direct and indirect effects of sleep attitudes on sleep outcomes. This hypothesis was partially supported, depending on the moderator variable tested. A breakdown of these analyses are provided below.

**Age.** PROCESS indicated the moderated mediation effect at three ages: the median age of 31, and 25 and 42 years old (+/− 1 SD). While younger individuals tended to have a larger effect size as compared to older individuals for both sleep duration and quality, there was no significant moderated mediation effect for age.

**Gender.** A significant moderated mediation effect was observed for gender in predicting sleep quality (CI [.14, 1.37]), with males (*b* = −1.49) having a larger effect size than females (*b* =  = −.78). Although the moderated mediation effect was not significant for sleep duration (CI [−.01, .20]), the same pattern was observed such that there was a larger effect size for male (*b* = −.15) than females (*b* = −.07). The indirect effect of sleep attitudes on sleep quality via sleep hygiene practices was most pronounced among males.

**Race.** The index of moderated mediation for race was not significant for sleep duration (CI [−.10, .09]) or sleep quality (CI [−.59, .75]). Effects sizes were similar for whites (*b* *=* −.13 and *b* *=* −1.2 for sleep duration and quality, respectively) and racial minorities (*b* = −.14 and *b* = −1.3 for sleep duration and quality, respectively).

**SES.** Similarly to age, PROCESS broke individuals’ perceived SES into thirds marked at 3, the median of 5, and 7. A significant moderated mediation effect was found for SES in predicting both sleep duration (*b* = −.02, CI [−.05, −.001]) and sleep quality (*b* = −.17, CI [−.31, −.01]), with the strongest effect observed for those of higher perceived SES. In other words, participants with higher perceived SES status showed the strongest indirect association of sleep attitudes on sleep duration and quality via sleep hygiene practices.

## Discussion

The present study hypothesized that (1) sleep attitudes would be significantly associated with both sleep duration and sleep quality indirectly via sleep hygiene practices among adults with an average age above 30, and, (2) the indirect effect of sleep hygiene would be contingent on varying levels of the demographic characteristics (i.e. age, gender, race, and SES), producing a moderated mediation effect. Hypothesis one was confirmed for both sleep duration and sleep quality, indicating that the relationship between sleep attitudes and sleep duration and quality operates indirectly via sleep hygiene practices. This extended the external validity of prior research demonstrating the same effect only in a more diverse, older sample of adults instead of college students alone. Lastly, results partially supported hypothesis two, indicating that the relationship between sleep attitudes and sleep quality via sleep hygiene varies by gender and SES. Only SES produced the moderated mediation effect for sleep duration. While gender was not significant for sleep duration, a similar pattern as sleep quality existed. Specifically, these relationships are the strongest for those who identify as men and for those of higher SES. The pattern observed for SES in the present study is consistent with prior research demonstrating that SES can be a better predictor of health disparities than race alone (e.g. Williams, 1996).

It is possible that the findings regarding gender could be linked to the idea of controllability. Adult women tend to have more responsibilities than men during the evening hours (i.e. engaging in childcare, taking care of the home) and women are more likely to become single parents, serve as a caregiver to an aging spouse or parent, and be widowed (Rieker & Bird, 2005). Therefore, women may de-prioritize their own health needs to tend to their children, parents, or spouse and therefore may have less control over their evening routine, including sleep (Hart, 2014). In comparison, men may have more discretionary time in the evenings to devote to sleep if they so choose. If this is the case, then the role of sleep attitudes on sleep practices may be more evident as a result of more freedom to choose.

The issue of control may also help explain the results found regarding SES. It is impossible to talk about group differences, such as gender or SES differences, without factoring in the mechanisms regarding what is actually *happening* for these groups that contribute to these differences in sleep attitudes and sleep outcomes. For instance, it is possible that the construct of SES in this study, as measured via self-reported subjective social status, is serving as a proxy variable for factors such as neighborhood safety, air quality both in the home and in the neighborhood, level of neighborhood noise, and even quality of pillows and mattresses to promote sleep. Therefore, perhaps instead of the mechanism being SES, it is actually the privilege associated with a higher SES, such as the privilege of having more control as a result of having more discretionary time. Greater control over home environment and greater choice in time use may promote better sleep, especially among those who value sleep. Additionally, higher SES could also be an indicator of better access to good education and healthcare, where more of an emphasis is placed on maintaining healthy behaviors, including sleep. These are also important considerations to reflect on when thinking about how an optimal or suboptimal physical/social environment, as measured by perceived SES, impacts sleep attitudes and outcomes, and whether the environment shapes one’s sleep attitudes (and thereby sleep outcomes), or whether one’s sleep attitudes exist first. For instance, neighborhood safety, which is related to socioeconomic status, has been linked with sleep outcomes (Fuller-Rowell et al., 2016), and could shape one’s attitudes toward sleep.

While race did not significantly moderate the relationship between sleep attitudes and sleep outcomes via sleep hygiene in the present study, it is possible that our measure of SES was also tapping into race since the two are likely conflated (Jackson & Williams, 2006). Furthermore, racial disparities in sleep are in part largely due to modifiable, non-genetic factors, such as discrimination, since race is socially constructed (Cooper & David, 1986). Discrimination then contributes to income, neighborhood/housing, quality of education, access to healthcare services, social capital, and public safety, in addition to other factors, which all contribute to disparities in sleep as well as subsequent disease and mortality (Williams & Jackson, 2005). Therefore, race acts as a proxy for, rather than a measure of, relative social advantage/disadvantage. It is important that future research prioritizes teasing apart the umbrella constructs of race and SES to examine the factors that make them up and are contributing directly to differences seen in sleep attitudes and sleep outcomes.

### Strengths and Limitations

Previous work involving sleep attitudes has focused primarily on college students (Peach & Gaultney, 2017). A strength of the present study is that it broadened the consideration of sleep attitudes to a sample representing a wider age range, as well as prior research demonstrating an indirect relationship between sleep attitudes and sleep outcomes via sleep hygiene in a sample of college students (Peach et al., 2018). However, this study was also a secondary data analysis used to extend and build on these previous findings, so future research should prioritize replicating these findings in a new, independent sample, to continue to build the external validity of these findings.

Additionally, the nature of this cross-sectional, self-reported and exploratory data means that any conclusions drawn here are tentative and require further support. Because all variables were measured at the same time, sleep hygiene can be viewed as an indirect effect but not a true mediator. Longitudinal data are needed to better understand directionality. A potential limitation worth mentioning again is that the demographic variables measured during this study could reflect group differences that have nothing to do with gender or SES, and more to do with differences in the freedom and control to make choices about when and how much one sleeps.

Additionally, the way in which race was dummy coded into two groups, consisting of White and racial minority, is a limitation. Given the relatively nascent nature of this line of research, categorizing the racial groups in this manner was a way to begin to understand how sleep attitudes and outcomes vary based on racial group. However, a review by Johnson, Jackson, Williams, and Alcantara (2019) examined selected sleep characteristics among racial/ethnic minorities compared to white individuals in the United States and found comparable differences in the racial minority groups (Black/African American, Hispanic/Latino, Asian, Native Hawaiian/Pacific Islander, Native American/Alaskan Native, and Multiracial/Other) as compared with whites. For instance, for both measures of sleep duration and sleep quality, it was found that all racial minority groups fell below the recommended amount of sleep duration and experienced poorer sleep quality compared to whites. Therefore, although future research should explore the between and within group differences of racial minority groups, prior research conducted on sleep outcomes in whites and racial minorities justifies dichotomizing these groups, at least for beginning investigations.

### Implications and conclusions

These secondary data extend previous work on sleep attitudes (Peach & Gaultney, 2017) by examining whether demographic-related differences in sleep attitudes exist and whether demographic differences in sleep attitudes predicted sleep outcomes indirectly via sleep hygiene practices. The results of the present study support the notion that health outcome interventions (such as sleep outcome interventions) should be tailored to the characteristics of the specific individual being targeted. If sleep attitudes are potentially modifiable health behaviors, it will be important to know whether the importance placed on assessing and addressing sleep attitudes in future sleep interventions should be differentially emphasized for different demographic groups. This is in line with the patient-centered care approach to healthcare, which focuses on the individual’s specific health needs and desired health outcomes, shaped by individual-level factors (Epstein & Street, 2011). Implementing an individualized standard of care is important considering some disenfranchized demographic groups experience disparities in overall health (e.g. Williams et al., 2010), as well as sleep outcomes (e.g. Williams et al., 2016). It is possible that sleep attitudes are a potentially modifiable prevention strategy for improving sleep and health more broadly, and future research should continue to explore how the impact of sleep attitudes on sleep outcomes, especially according to an individual’s demographic characteristics.

## Data Availability

The authors plan to provide full publicly available access to the present study’s data should the paper be accepted for publication.
